# PROK2/PROKR2 Signaling and Kallmann Syndrome

**DOI:** 10.3389/fendo.2013.00019

**Published:** 2013-04-12

**Authors:** Catherine Dodé, Philippe Rondard

**Affiliations:** ^1^INSERM U1016, Institut Cochin, Université Paris-DescartesParis, France; ^2^CNRS UMR5203, INSERM U661, Institut de Génomique Fonctionnelle, Université Montpellier 1, 2Montpellier, France

**Keywords:** PROK2, PROKR2, Kallmann syndrome, hypogonadotropic hypogonadism, anosmia, digenic/oligogenic mode of inheritance

## Abstract

Kallmann syndrome (KS) is a developmental disease that associates hypogonadism and a deficiency of the sense of smell. The reproductive phenotype of KS results from the primary interruption of the olfactory, vomeronasal, and terminal nerve fibers in the frontonasal region, which in turn disrupts the embryonic migration of neuroendocrine gonadotropin-releasing hormone (GnRH) synthesizing cells from the nose to the brain. This is a highly heterogeneous genetic disease, and mutations in any of the nine genes identified so far have been found in approximately 30% of the KS patients. *PROKR2* and *PROK2*, which encode the G protein-coupled prokineticin receptor-2 and its ligand prokineticin-2, respectively, are two of these genes. Homozygous knockout mice for the orthologous genes exhibit a phenotype reminiscent of the KS features, but biallelic mutations in *PROKR2* or *PROK2* (autosomal recessive mode of disease transmission) have been found only in a minority of the patients, whereas most patients carrying mutations in these genes are heterozygotes. The mutations, mainly missense mutations, have deleterious effects on PROKR2 signaling in transfected cells, ranging from defective cell surface-targeting of the receptor to defective coupling to G proteins or impaired receptor-ligand interaction, but the same mutations have also been found in apparently unaffected individuals, which suggests a digenic/oligogenic mode of inheritance of the disease in heterozygous patients. This non-Mendelian mode of inheritance has so far been confirmed only in a few patients. However, it may account for the unusually high proportion of KS sporadic cases compared to familial cases.

## Introduction

Kallmann syndrome (KS) is a developmental disease that associates hypogonadotropic hypogonadism, due to gonadotropin-releasing hormone (GnRH) deficiency, and anosmia, related to the absence or hypoplasia of the olfactory bulbs (Kallmann, 1944; deMorsier, 1954; Naftolin et al., 1971). The degree of the hypogonadism and that of the smell deficiency can vary significantly, not only between unrelated KS patients, but also between patients from the same family. The prevalence of KS has been estimated at one out of 8000 in boys. In girls, the prevalence is thought to be five times lower, but it is probably underestimated because some affected females only have mild hypogonadism, and also because primary amenorrhea in females often remains unexplored (Jones and Kemmann, 1976).

Pathohistological studies of fetuses with olfactory bulb agenesis have shown that the reproductive phenotype of KS results from a pathological sequence in embryonic life, whereby premature interruption of the olfactory, vomeronasal, and terminal nerve fibers in the frontonasal region disrupts the migration of neuroendocrine GnRH cells, which normally migrate from the nose to the brain along these nerve fibers (Schwanzel-Fukuda and Pfaff, 1989; Teixeira et al., 2010). What causes the primary failure of these fibers to establish proper contact with the forebrain is, however, still unknown.

Kallmann syndrome is genetically heterogeneous and involves various modes of transmission, specifically, X-chromosome linked, autosomal recessive, autosomal dominant with incomplete penetrance, and also digenic/oligogenic inheritance (Dodé and Hardelin, 2009; Sykiotis et al., 2010). Because the common infertility in affected individuals and, most importantly, the incomplete penetrance of the disease impede genetic linkage analysis, researchers have used various strategies to identify genes involved in KS, including mutation screening in genes that are disrupted by deletion or translocation breakpoints in chromosomal rearrangements associated with the disease phenotype, and candidate gene approaches. Nine causal genes have been reported to date, namely, by chronological order of discovery, *KAL1* (Franco et al., 1991; Legouis et al., 1991; Hardelin et al., 1992), *FGFR1* (Dodé et al., 2003), *PROKR2* and *PROK2* (Dodé et al., 2006), *FGF8* (Falardeau et al., 2008), *CHD7* (Kim et al., 2008; Jongmans et al., 2009), *WDR11* (Kim et al., 2010), *HS6ST1* (Tornberg et al., 2011), and *SEMA3A* (Hanchate et al., 2012; Young et al., 2012) (Table 1). Various loss-of-function mutations in *KAL1*, encoding the extracellular matrix glycoprotein anosmin-1, and in *FGFR1* or *FGF8*, encoding fibroblast growth factor receptor-1 and fibroblast growth factor-8, underlie the X-chromosome linked form and an autosomal dominant form of KS, respectively. Mutations in *KAL1* and *FGFR1/FGF8* account for roughly 8 and 10% of all KS cases, respectively. The *KAL1* gene product, anosmin-1, binds to heparan-sulfate glycosaminoglycans, and may act as a co-receptor for FGF signaling through FGFR1, which also requires interaction with heparan-sulfate glycosaminoglycans for receptor activation. Mutations in the genes encoding heparan-sulfate 6-*O*-sulfotransferase 1, an enzyme involved in glycosaminoglycan modifications, WDR11, an intracellular protein that interacts with the transcription factor EMX1, and semaphorin 3A, a secreted protein involved in axonal pathfinding, have also been found in some KS patients. Mutations in the chromodomain helicase DNA-binding protein 7 gene (*CHD7*) are present in approximately 70% of the patients affected by the CHARGE syndrome, which in most patients includes KS (Pinto et al., 2005), and mutations in this gene have been found in some patients who initially presented with KS. Finally, mutations in *PROKR2* and *PROK2*, encoding prokineticin receptor-2 and prokineticin-2, respectively, have been identified in approximately 9% of the KS patients, both in heterozygous and in homozygous or compound heterozygous states.

**Table 1:** **KS genes**.

Genes	*KAL1*	*FGFR1* and *FGF8*	*PROKR2* and *PROK2*	*CHD7*	*WDR11*	*HS6ST1*	*SEMA3A*
Mode(s) of transmission	X-linked recessive	Autosomal dominant (incomplete penetrance) or digenic/oligogenic	Autosomal recessive or digenic/oligogenic	?	?	digenic/oligogenic?	digenic/oligogenic?
Strategy for gene identification	Cytogenetics	Cytogenetics and mouse model	Mouse models	Candidate gene	Cytogenetics	*C. elegans* model	Mouse model
Prevalence of mutations in KS patients	8% of male patients	10 and <1%	7 and 3%	1–5%	<1%	<1%	6%
Reference	Legouis et al. (1991), Franco et al. (1991), Hardelin et al. (1992)	Dodé et al. (2003), Falardeau et al. (2008)	Dodé et al. (2006)	Kim et al. (2008), Jongmans et al. (2009)	Kim et al. (2010)	Tornberg et al. (2011)	Hanchate et al. (2012), Young et al. (2012)

*?, not known*.

## Prokineticins and Their Receptors

Prokineticins are secreted cysteine-rich proteins that possess diverse biological activities. The first identified member of this family was isolated from the venom of the black mamba snake (Joubert and Strydom, 1980), and was named mamba intestinal toxin 1 (MIT1) owing to its ability to induce intestinal contraction (Schweitz et al., 1999). Then, a small protein of similar size (77 amino acid residues, 8 kDa), with 58% sequence identity with MIT1, was isolated from skin secretions in the amphibian *Bombina variegata*, and called Bv8 (Mollay et al., 1999). Soon after, two mammalian proteins of this family were identified and named prokineticin-1 and -2 (PROK1 and PROK2) (Li et al., 2001; Kaser et al., 2003). PROK1 has 80% sequence identity with MIT1 and 58% identity with PROK2 (Li et al., 2001). The amino-terminal domain of prokineticins contains a sequence of six amino acid residues (AVITGA), which is conserved in all mammalian and non-mammalian orthologs. Substitutions, deletions, or insertions to this hexapeptide result in the loss of agonist activity on prokineticin receptors (Kaser et al., 2003; Bullock et al., 2004). The carboxy-terminal region of prokineticins contains 10 cysteine residues forming five disulfide bonds. Apart from its potent effect on gastrointestinal smooth muscle contraction, PROK1 was also characterized as an angiogenic factor with specific effects on steroidogenic glands, thus earning its initial name EG-VEGF (endocrine gland vascular endothelial growth factor) (LeCouter et al., 2003). Mouse and human orthologs of Bv8, also known as PROK2, have been involved in a variety of biological activities, including effects on neuronal survival, gastrointestinal smooth muscle contraction (Li et al., 2001), circadian locomotor rhythm (Cheng et al., 2002), survival and migration of adrenal cortical capillary endothelial cells (LeCouter et al., 2003). PROK2 also has a role in appetite regulation and its anorectic effect is mediated partly by the melanocortin system (Gardiner et al., 2006).

Prokineticins can bind to two different G protein-coupled receptors, prokineticin receptor-1 and -2 (PROKR1 and PROKR2), which have about 85% sequence identity. These receptors were characterized simultaneously by three different groups (Lin et al., 2002; Masuda et al., 2002; Soga et al., 2002). Both are able to bind to PROK1 and PROK2 with similar nanomolar range affinities. They have a central core formed by seven transmembrane α-helical segments (TM1–TM7) connected by intracellular (i1–i3) and extracellular (e1–e3) loops, an extracellular amino-terminal end, and an intracellular carboxy-terminal end. These plasma membrane receptors operate as molecular switches to relay extracellular ligand-activation to intracellular heterotrimeric G proteins. PROKR1 is mainly expressed in peripheral tissues, including endocrine glands and organs of the reproductive system, the gastrointestinal tract, lungs, and the circulatory system (Soga et al., 2002; Battersby et al., 2004), whereas PROKR2 shows relatively localized distribution in the central nervous system (Cheng et al., 2002; Lin et al., 2002).

The olfactory bulb is one of the few areas in the mammalian brain that produce neurons throughout life. New interneurons originating from progenitors in the subventricular zone (SVZ) are continually added to the olfactory bulbs. The mRNAs of both receptors (PROKR1 and PROKR2) are expressed in the SVZ and the olfactory bulbs. The expression of PROKR1 have been detected in these areas although less abundantly than PROKR2. Whereas PROK1 mRNAs were not detected in any of these brain region (Ng et al., 2005). It was first shown that PROK2 functions as a chemoattractant for these neuronal progenitors, which follow a rostral migratory stream. Accordingly, PROK2 deficiency in mice leads to a loss of normal olfactory bulb architecture, and accumulation of neuronal progenitors in the rostral migratory stream (Ng et al., 2005). Soon after, it was found that *Prokr2^−/−^* knockout mice exhibit early hypoplasia of the olfactory bulbs and severe atrophy of the reproductive organs in both sexes, a phenotype reminiscent of the KS features. In addition, immunohistochemical analysis of these mice revealed that the neuroendocrine GnRH cells were absent from the hypothalamus (Matsumoto et al., 2006).

*PROK2* is a clock-controlled gene: the level of its messenger RNA shows a circadian oscillation profile in the suprachiasmatic nuclei (Cheng et al., 2002; Li et al., 2006). It has been postulated that PROK2 signaling through PROKR2 is a suprachiasmatic nuclei clock output signal that regulates circadian rhythms (Prosser et al., 2007; Li et al., 2012). PROK2-null mice show accelerated acquisition of food anticipatory activity during a daytime food restriction (Li et al., 2006), exhibit reduced total sleep time predominantly during the light period, and also have an impaired response to sleep disturbance (Hu et al., 2007).

PROK2 is a functional target gene of proneural basic helix-loop-helix (bHLH) factors. Neurogenin-1 (NGN1) and MASH1 activate *PROK2* transcription by binding to E-box motifs on the *PROK2* promoter with the same set of E-boxes critical for another pair of bHLH factors, CLOCK and BMAL1, in the regulation of circadian clock (Cheng et al., 2002; Zhang et al., 2007).

## Complex Genetics of Kallmann Syndrome Caused by Mutations in *PROKR2* or *PROK2*

We first considered that *PROKR2* was a relevant KS candidate because of the KS-like phenotype of PROKR2-null mice (see above). We thus sequenced the two coding exons of *PROKR2* in a cohort of patients affected by KS, and identified 10 different mutations (one frame-shifting and nine missense mutations) in 14 patients, either in heterozygous state (10 cases) or in homozygous or compound heterozygous state (4 cases) (Dodé et al., 2006) (Table 2). Notably, most of these mutations were missense mutations, and many were also found in apparently unaffected individuals, thus initially raising some questions regarding their pathogenic role. A deleterious effect on the signaling activity of PROKR2 was, however, confirmed in transfected HEK-293 cells for most of the mutations (Cole et al., 2008; Monnier et al., 2009).

**Table 2:** ***PROKR2* and *PROK2* mutations in Kallmann syndrome**.

Exon	Nucleotide change	Amino acid change	Localization (domain)	Functional consequence	Reference
***PROKR2***					
1	58del	Frameshift	N-terminal region	NMD or protein truncation	Dodé et al. (2006)
	151G > A	A51T	N-terminal region	None?	Reynaud et al. (2012), Sbai et al. (unpublished)
	238C > T	R80C	First intracellular loop	None?	Abreu et al. (2010), Sbai et al. (unpublished)
–	253C > T	R85C	–	Mild G protein-coupling defect	Cole et al. (2008), Monnier et al. (2009), Sarfati et al. (2010)
–	253C > G	R85G	–	Strong G protein-coupling defect	Sarfati et al. (2010), Raivio et al. (2012)
–	254G > T	R85L	–	Mild protein-coupling defect	Sarfati et al. (2010), Sbai et al. (unpublished)
–	254G > A	R85H	–	Mild G protein-coupling defect	Dodé et al. (2006), Monnier et al. (2009)
–	337T > C	Y113H	First extracellular loop	Strong G protein-coupling defect	Cole et al. (2008), Sbai et al. (unpublished)
–	343G > A	V115M	–	Strong G protein-coupling defect	Cole et al. (2008), Sbai et al. (unpublished)
	349C > T	R117W	–	Strong G protein-coupling defect	Sbai et al. (unpublished)
–	420C > G	Y140X	Third transmembrane domain	NMD or protein truncation	Abreu et al. (2010)
2	491G > A	R164Q	Second intracellular loop	Mild G protein-coupling defect	Dodé et al. (2006), Cole et al. (2008), Monnier et al. (2009)
–	518T > G	L173R	Fourth transmembrane domain	Cell surface-targeting defect	Dodé et al. (2006), Abreu et al. (2010), Cole et al. (2008), Monnier et al. (2009)
–	533G > C	W178S	–	Cell surface-targeting defect	Dodé et al. (2006), Cole et al. (2008), Monnier et al. (2009)
–	563C > T	S188L	–	Strong G protein-coupling defect	Cole et al. (2008), Sbai et al. (unpublished)
–	604A > G	S202G	Second extracellular loop	?	Chan et al. (2009)
–	629A > G	Q210R	–	Ligand-binding defect	Dodé et al. (2006), Monnier et al. (2009)
	701G > A	G234D	Fifth transmembrane domain	Cell surface-targeting defect	Sbai et al. (unpublished)
–	743G > A	R248Q	Third intracellular loop	None?	Cole et al. (2008)
–	752G > T	W251L	–	Strong G protein-coupling defect?	Sarfati et al. (2010), Sbai et al. (unpublished)
–	802C > T	R268C	–	G protein-coupling defect?	Dodé et al. (2006), Abreu et al. (2010), Monnier et al. (2009), Sarfati et al. (2010)
–	T820 > A	V274D	Sixth transmembrane domain	Strong G protein-coupling defect	Sinisi et al. (2008), Sbai et al. (unpublished)
–	868C > T	P290S	–	Cell surface-targeting defect	Dodé et al. (2006), Monnier et al. (2009)
–	969G > A	M323I	Seventh transmembrane domain	None?	Dodé et al. (2006), Monnier et al. (2009)
–	989del	Frameshift	–	NMD or protein truncation	Sarfati et al. (2010)
–	991G > A	V331M	–	Mild G protein-coupling defect	Dodé et al. (2006), Cole et al. (2008), Monnier et al. (2009), Sarfati et al. (2010)
–	1069C > T	R357W	C-terminal region	None?	Cole et al. (2008), Sbai et al. (unpublished)
***PROK2***					
1	−4C > A		Translation initiation site	Reduced protein synthesis?	Dodé et al. (2006)
–	70G > C	A24P	Signal peptide	?	Cole et al. (2008)
–	94G > C	G32R	AVITGA motif	Strongly impaired activity?	Dodé et al. (2006)
2	101 G > A	C34Y	Cysteine-rich region	Strongly impaired activity	Cole et al. (2008)
–	137G > A	C46Y	Cysteine-rich region	?	Dodé et al. (unpublished)
–	150C > G	I50M	–	None?	Cole et al. (2008)
–	161G > A	S54N	–	?	Sarfati et al. (2010)
–	163del	Frameshift	–	NMD or protein truncation	Pitteloud et al. (2007), Leroy et al. (2008)
–	217C > T	R73C	–	Strongly impaired activity	Dodé et al. (2006), Leroy et al. (2008), Cole et al. (2008)
4	297_298insT	Frameshift	–	NMD or protein truncation	Dodé et al. (2006), Abreu et al. (2010)
	301C > T	R101W	–	?	Dodé et al. (unpublished)
–	302G > A	R101Q	–	?	Dodé et al. (unpublished)
–	310C > T	H104Y	–	?	Sarfati et al. (2010)

*Mutations reported in *PROKR2* and *PROK2* are mainly missense mutations. In most patients, the mutations have been found in heterozygous state. The R85C, R85H, R164Q, L173R, and P290S *PROKR2* mutations, as well as R73C, c.163del, and c.297_298insT *PROK2* mutations have, however, been found both in heterozygous and homozygous (or compound heterozygous) states, which suggests that patients heterozygous for *PROKR2* or *PROK2* mutations carry additional mutations, presumably in other, as yet unidentified Kallmann syndrome genes in most cases. Notably, two such patients have the L173R mutation in *PROKR2* together with S396L or R423X mutations in *KAL1* (Dodé et al., 2006; Sarfati et al., 2010), another patient has the V115M mutation in *PROKR2* together with the A24P mutation in *PROK2* (Cole et al., 2008), and still another patient has the R85L mutation in *PROKR2* together with a A604T mutation in *FGFR1* (Sarfati et al., 2010). In addition, the patient who has the S202G mutation in *PROKR2* also has I239T and R31C monoallelic mutations in *FGFR1* and *GNRH1*, respectively (Chan et al., 2009). Finally, two patients who carry R268C and V331M mutations in *PROKR2* also carry A189T and R240Q monoallelic mutations in *KISS1R* and *GNRHR*, respectively (Sarfati et al., 2010). Abbreviation: NMD, nonsense-mediated mRNA decay*.

*?, not known*.

**Figure 1:**
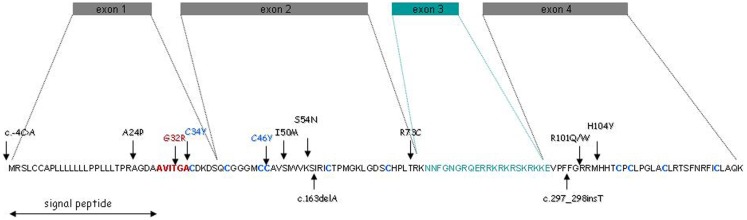
**PROK2 gene structure and amino acid sequence**. The green box and green amino acid sequence denote the alternative exon and encoded peptide, respectively. The AVITGA motif is shown in red, and the 10 cysteinyl residues (forming five disulfide bonds) are in blue. Vertical arrows indicate the positions of the mutations identified in the patients.

Then, we considered the possibility that mutations in *PROK2* also account for some KS cases, especially since mutant mice defective in PROK2 showed a marked reduction in the size of their olfactory bulbs. *PROK2* contains four coding exons (Bullock et al., 2004), of which exon 3, encoding an arginine- and lysine-rich peptide of 21 amino acid residues, may or may not be included in the mature transcript due to alternative splicing (Wechselberger et al., 1999) (Figure 1). We sequenced the entire coding sequence of *PROK2* in the patients, and identified four different point mutations (two missense mutations, one frame-shifting mutation, and one single nucleotide substitution in the translation initiation sequence), all in the heterozygous state (Dodé et al., 2006). *PROK2* mutations in homozygous state were subsequently found in a few patients, and a KS-like phenotype was concomitantly reported in PROK2-null mutant mice (Pitteloud et al., 2007; Leroy et al., 2008). Since then, additional mutations in *PROKR2* and *PROK2* have been reported in KS patients. A list of the mutations, together with the corresponding references, is provided in Table 2.

The finding, for given *PROKR2* and *PROK2* mutations, of both heterozygous and homozygous (or compound heterozygous) unrelated patients is quite remarkable, and argues in favor of a digenic or oligogenic mode of inheritance in heterozygous patients. To date, digenic inheritance of KS has been shown in few patients who had monoallelic missense mutations both in *PROKR2* or *PROK2*, and in other KS genes (*KAL1*, *FGFR1*) or genes underlying normosmic congenital hypogonadotropic hypogonadism (*GNRHR*, *KISS1R*) (Dodé et al., 2006; Cole et al., 2008; Raivio et al., 2009; Martin et al., 2010; Sarfati et al., 2010). The other patients harboring monoallelic mutations in *PROKR2* or *PROK2* are expected to carry at least one additional pathogenic mutation in as yet uncharacterized genes. Indeed, mutations in any of the currently known KS genes have been identified in only 30% of all KS patients, thus indicating that other disease genes remain to be discovered. Notably, it has been found that patients carrying biallelic mutations in *PROKR2* or *PROK2* consistently have a severe, complete KS phenotype, whereas the phenotype of patients carrying monoallelic mutations in these genes is more variable, and likely depends on the additional genetic hits in these patients (Martin et al., 2010; Sarfati et al., 2010).

Kallmann syndrome patients who carry biallelic mutations in *PROK2* or *PROKR2* do not seem to have any of the non-olfactory, non-reproductive occasional anomalies that have been reported in the previously characterized genetic forms of the disease (i.e., X-linked KAL1 form and autosomal dominant KAL2 form), specifically, bimanual synkinesis, renal agenesis, dental agenesis, and cleft lip or palate, and even though sleep disorders or increased body mass index have been reported in some of these patients, there is so far no evidence that these clinical features can be ascribed to the *PROKR2* and *PROK2* mutations, despite potential roles of these genes in sleep-wake regulation and ingestive behavior (see above). In addition, plasma cortisol levels were measured during 24 h in five patients mutated in *PROK2* or *PROKR2*, including one patient with biallelic *PROKR2* mutations, and normal circadian variation was observed in all cases (Sarfati et al., 2010), which argues against a major contribution of PROK2/PROKR2 signaling to physiological circadian variation of plasma cortisol levels in humans. Of course, this result does not exclude the presence of more subtle defects in the patients.

## Consequences of the *PROKR2* and *PROK2* Missense Mutations on Receptor Signaling Activity

**Figure 2:**
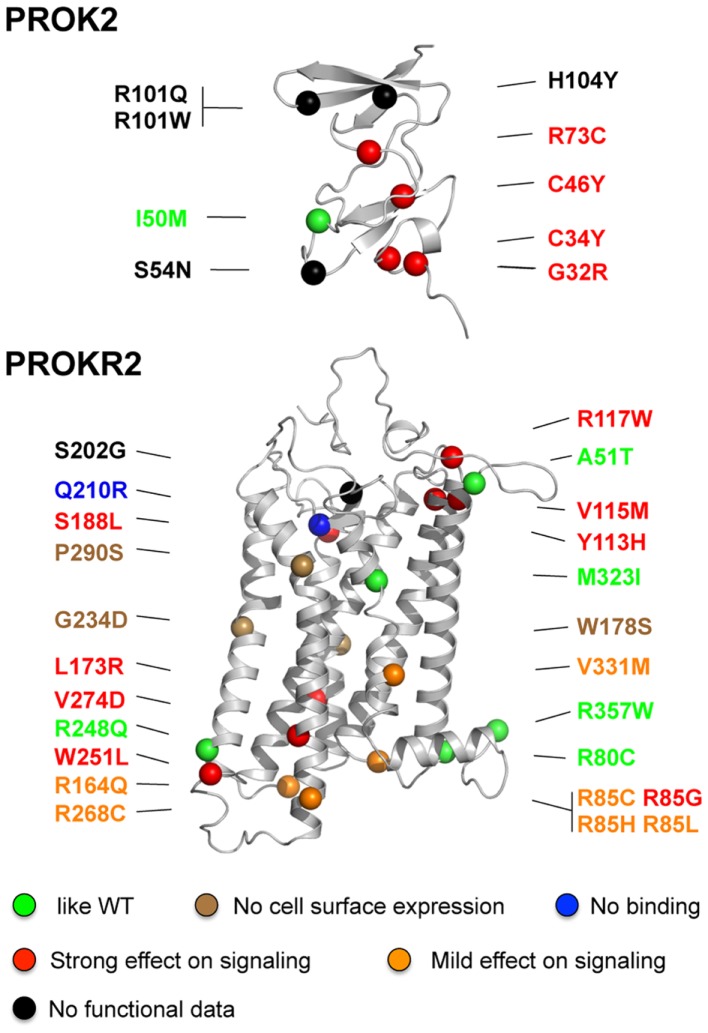
**Positions of missense mutations in PROK2 and PROKR2 in the structural models of the ligand and the receptor**. The mutations are classified in different categories according to their effects on PROKR2 signaling activity: similar to wild-type (green), absence of the receptor at the cell surface (brown), absence of ligand-binding (blue), and strong or mild effect on signaling (red and orange, respectively). The mutations for which functional data are not available are denoted in black. The colored balls indicate the atom of alpha carbon of the polypeptidic chain of the mutated residue. Note that residue R357 is located in the proximal part of the C-terminal region of the receptor.

A total of 24 *PROKR2* missense mutations have been identified (Dodé et al., 2006; Cole et al., 2008; Sinisi et al., 2008; Chan et al., 2009; Abreu et al., 2010; Sarfati et al., 2010) in KS patients (Table 2; Figure 2). Most of the mutant receptors have been characterized regarding their ability to induce intracellular release of calcium upon PROK2-stimulation, their cell surface expression, and their PROK2-binding, in mammalian cell lines (Cole et al., 2008; Monnier et al., 2009; Abreu et al., 2012; Raivio et al., 2012; Sbai et al., in preparation). Although PROKR2 can activate different G protein pathways (Lin et al., 2002; Soga et al., 2002; Chen et al., 2005), its coupling to Gq, leading to intracellular release of calcium, represents the best characterized transduction mechanism. Only five mutants (A51T, R80C, R248Q, M321I, R357W) have properties similar to the wild-type PROKR2, thus calling into question the pathogenic effect of these missense variants. Notably, the R268C mutation has also been found in heterozygous state in a relatively large proportion (174 out of 2203, i.e., 7.9%) of individuals from the African American general population, and in homozygous state in six individuals from the same population (0.3%; see Exome Variant Server website URL: http://evs.gs.washington.edu/EVS/). Moreover, a clear deleterious effect of the R268C mutation on PROKR2 signaling through Gq protein activation could not be found in transfected HEK-293 cells, thus calling into question the pathogenic effect of this missense variant too. Most of the *PROKR2* mutations, however, impair cell surface expression, ligand-binding, or G protein-binding of the receptor (Figure 2). Three mutations affecting conserved residues located in the middle of the transmembrane helices, W178S, G234D, and P290S, impede targeting of the receptor to the cell surface. The Q210R mutant receptor is present at the cell surface, but is not able to bind to the ligand. The other mutants impair, either mildly or strongly, intracellular release of calcium in the Gq signaling pathway, and for some of them this effect might be due to the low expression of the mutant at the cell surface. Interestingly, the mutations that mostly impair the signaling are located nearby the extracellular side or the intracellular side of the receptor, and for these mutants, the loss-of-function is associated to a loss of cell surface expression. The mutations that result in a mildly impaired signaling activity are located in the intracellular loops, in agreement with the important role played by these loops in G protein-coupling. In addition, when wild-type and mutant receptors were coexpressed in HEK-293 cells, none of the mutant receptors that were retained within the cells did affect cell surface-targeting of the wild-type receptor, and none of the mutant receptors properly addressed at the plasma membrane did affect wild-type receptor signaling activity. This argues against a dominant negative effect of the mutations *in vivo*.

A total of 10 missense mutations have been identified in PROK2 (Table 2, Figure 2) (Dodé et al., 2006; Cole et al., 2008; Leroy et al., 2008; Sarfati et al., 2010). A mutation located at the translation initiation site (−4C > A) likely reduced the protein synthesis. Mutations of conserved residues in prokineticins are expected to strongly impair PROK2 activity such as those in the N-terminal conserved region AVITGA important for prokineticin activity (G32R), and those of the conserved cysteines (C34Y and C46Y). In addition, mutations that introduce an additional cysteine (R73C) in the cysteine-rich region of PROK2 may affect folding of the hormone then leading to a loss-of-function. This is consistent with the functional analysis of Cole et al. (2008) who have examined the signaling properties of three PROK2 mutants: the C34Y and R73C mutations totally abolished and strongly impaired the intracellular calcium release activity of PROKR2, respectively, whereas the I50M mutation had an activity similar to that of the wild-type PROK2. The functional consequence of the other mutations (A24P, S54N, R101Q, R101W, and H104Y) are more difficult to predict in the absence of model of the complex between PROK2 and PROKR2. Further studies will be necessary to better analyze whether the mutations in PROK2 affect the process of binding and/or activation of PROKR2 by the hormone.

## Conclusion

The characterization of new genes involved in KS is a difficult goal. Because of the hypogonadotropic hypogonadism, sizes of the KS families are small and the mode of inheritance is very often difficult to establish. Animal models in which a gene has been inactivated and involving a KS phenotype are an alternative approach for the identification of new KS genes. The inactivation of *PROK2* or *PROKR2* lead to defective olfactory morphogenesis and hypogonadism in mice and humans. Curiously, *PROK2* and *PROKR2* mutations in homozygous state were found in a few patients and the main part of the KS patients carried only heterozygous mutations. In all of the functional studies done so far, only Gq signaling pathways have been investigated to characterize the PROK2 and PROKR2 mutants and further studies will be necessary to analyze other signaling pathways. Interestingly, few patients who had monoallelic missense mutations both in *PROK2* or *PROKR2*, and in other KS or normosmic congenital hypogonadotropic hypogonadism genes, raising the idea of oligogenism. So far, this hypothesis has been validated only in a few patients suggesting that the second mutation in the other heterozygous patients resides in unexplored regions of the genes or as yet undiscovered KS genes.

## Conflict of Interest Statement

The authors declare that the research was conducted in the absence of any commercial or financial relationships that could be construed as a potential conflict of interest.

## References

[B1] Abreu A. P. Kaiser U. B. Latronico A. C. (2010). The role of prokineticins in the pathogenesis of hypogonadotropic hypogonadism. Neuroendocrinology 91, 283–290 10.1159/000308880 20502053 PMC2968764

[B2] Abreu A. P. Noel S. D. Xu S. Carroll R. S. Latronico A. C. Kaiser U. B. (2012). Evidence of the importance of the first intracellular loop of prokineticin receptor 2 in receptor function. Mol. Endocrinol. 26, 1417–1427 10.1210/me.2012-1102 22745195 PMC3404297

[B3] Battersby S. Critchley H. O. Morgan K. Millar R. P. Jabbour H. N. (2004). Expression and regulation of the prokineticins (endocrine gland-derived vascular endothelial growth factor and Bv8) and their receptors in the human endometrium across the menstrual cycle. J. Clin. Endocrinol. Metab. 89, 2463–2469 10.1210/jc.2003-032012 15126578

[B4] Bullock C. M. Li J.-D. Zhou Q.-Y. (2004). Structural determinants required for the bioactivities of prokineticins and identification of prokineticin receptor antagonists. Mol. Pharmacol. 65, 582–588 10.1124/mol.65.3.582 14978236

[B5] Chan Y. M. de Guillebon A. Lang-Muritano M. Plummer L. Cerrato F. Tsiaras S. (2009). GNRH1 mutations in patients with idiopathic hypogonadotropic hypogonadism. Proc. Natl. Acad. Sci. U.S.A. 106, 11703–11708 10.1073/pnas.0812904106 19567835 PMC2710623

[B6] Chen J. Kuei C. Sutton S. Wilson S. Yu J. Kamme F. (2005). Identification and pharmacological characterization of prokineticin 2 β as a selective ligand for prokineticin receptor 1. Mol. Pharmacol. 67, 2070–2076 10.1124/mol.104.003814 15772293

[B7] Cheng M. Bullock C. M. Li C. Lee A. G. Bermak J. C. Belluzzi J. (2002). Prokineticin 2 transmits the behavioural circadian rhythm of the suprachiasmatic nucleus. Nature 417, 405–410 10.1038/417405a 12024206

[B8] Cole L. W. Sidis Y. Zhang C. Quinton R. Plummer L. Pignatelli D. (2008). Mutations in prokineticin 2 and prokineticin receptor 2 genes in human gonadotrophin-releasing hormone deficiency: molecular genetics and clinical spectrum. J. Clin. Endocrinol. Metab. 93, 3551–3559 10.1210/jc.2007-2654 18559922 PMC2567850

[B9] deMorsier G. (1954). Etudes sur les dysraphies crânio-encéphaliques. I. Agénésie des lobes olfactifs (telencéphaloschizis latéral) et des commissures calleuse et antérieure (telencéphaloschizis médian). La dysplasie olfacto-génitale. Schweiz. Arch. Neurol. Psychiatr. 74, 309–361 14385744

[B10] Dodé C. Hardelin J.-P. (2009). Kallmann syndrome. Eur. J. Hum. Genet. 17, 139–146 10.1038/ejhg.2008.206 18985070 PMC2986064

[B11] Dodé C. Levilliers J. Dupont J.-M. De Paepe A. Le Dû N. Soussi-Yanicostas N. (2003). Loss-of-function mutations in FGFR1 cause autosomal dominant Kallmann syndrome. Nat. Genet. 33, 463–465 10.1038/ng1122 12627230

[B12] Dodé C. Teixeira L. Levilliers J. Fouveaut C. Bouchard P. Kottler M.-L. (2006). Kallmann syndrome: mutations in the genes encoding prokineticin-2 and prokineticin receptor-2. PLoS Genet. 2:e175 10.1371/journal.pgen.0020175 17054399 PMC1617130

[B13] Falardeau J. Chung W. C. Beenken A. Raivio T. Plummer L. Sidis Y. (2008). Decreased FGF8 signaling causes deficiency of gonadotropin-releasing hormone in humans and mice. J. Clin. Invest. 118, 2822–2831 10.1172/JCI34538 18596921 PMC2441855

[B14] Franco B. Guioli S. Pragliola A. Incerti B. Bardoni B. Tonlorenzi R. (1991). A gene deleted in Kallmann’s syndrome shares homology with neural cell adhesion and axonal path-finding molecules. Nature 353, 529–536 10.1038/353529a0 1922361

[B15] Gardiner J. V. Bataveljic A. Patel N. A. Bewick G. A. Roy D. Campbell D. (2006). Prokineticin 2 is a hypothalamic neuropeptide that potently inhibits food intake. Diabetes 59, 397–406 10.2337/db09-1198 19933997 PMC2809973

[B16] Hanchate N. K. Giacobini P. Lhuillier P. Parkash J. Espy C. Fouveaut C. (2012). SEMA3A, a gene involved in axonal pathfinding, is mutated in some patients with Kallmann syndrome. PLoS Genet. 8:e1002896 10.1371/journal.pgen.1002896 22927827 PMC3426548

[B17] Hardelin J.-P. Levilliers J. del Castillo I. Cohen-Salmon M. Legouis R. Blanchard S. (1992). X chromosome-linked Kallmann syndrome: stop mutations validate the candidate gene. Proc. Natl. Acad. Sci. U.S.A. 89, 8190–8194 10.1073/pnas.89.17.8190 1518845 PMC49883

[B18] Hu W. P. Li J. D. Zhang C. Boehmer L. Siegel J. M. Zhou Q. Y. (2007). Altered circadian and homeostatic sleep regulation in prokineticin 2-deficient mice. Sleep 30, 247–256 17425220 PMC2673012

[B19] Jones J. Kemmann E. (1976). Olfacto-genital dysplasia in the female. Obstet. Gynecol. Annu. 5, 443 790240

[B20] Jongmans M. C. van Ravenswaaij-Arts C. M. Pitteloud N. Ogata T. Sato N. Claahsen-van der Grinten H. L. (2009). CHD7 mutations in patients initially diagnosed with Kallmann syndrome – the clinical overlap with CHARGE syndrome. Clin. Genet. 75, 65–71 10.1111/j.1399-0004.2008.01107.x 19021638 PMC2854009

[B21] Joubert F. J. Strydom D. J. (1980). Snake venom: the amino acid sequence of protein A from *Dendroaspis polylepis* (black mamba) venom. Hoppe-Seyler’s Z. Physiol. Chem. 361, 1787–1794 10.1515/bchm2.1980.361.1.425 7461607

[B22] Kallmann F. J. (1944). The genetic aspects of primary eunuchoidism. Am. J. Ment. Defic. 48, 203–236

[B23] Kaser A. Winklmayr M. Lepperdinger G. Kreil G. (2003). The AVIT protein family. EMBO Rep. 4, 469–473 10.1038/sj.embor.embor830 12728244 PMC1319185

[B24] Kim H. G. Ahn J.-W. Kurth I. Ullmann R. Kim H.-T. Kulharya A. (2010). WDR11, a WD protein that interacts with transcription factor EMX1, is mutated in idiopathic hypogonadotropic hypogonadism and Kallmann syndrome. Am. J. Hum. Genet. 87, 465–479 10.1016/j.ajhg.2010.08.018 20887964 PMC2948809

[B25] Kim H. G. Kurth I. Lan F. Meliciani I. Wenzel W. Eom S. H. (2008). Mutations in CHD7, encoding a chromatin-remodeling protein, cause idiopathic hypogonadotropic hypogonadism and Kallmann syndrome. Am. J. Hum. Genet. 83, 511–519 10.1016/j.ajhg.2008.09.005 18834967 PMC2561938

[B26] LeCouter J. Lin R. Tejada M. Frantz G. Peale F. Hillan K. J. (2003). The endocrine-gland-derived VEGF homologue Bv8 promotes angiogenesis in the testis: localization of Bv8 receptors to endothelial cells. Proc. Natl. Acad. Sci. U.S.A. 100, 2685–2690 10.1073/pnas.0337667100 12604792 PMC151401

[B27] Legouis R. Hardelin J.-P. Levilliers J. Claverie J.-M. Compain S. Wunderle V. (1991). The candidate gene for the X-linked Kallmann syndrome encodes a protein related to adhesion molecules. Cell 67, 423–435 10.1016/0092-8674(91)90193-3 1913827

[B28] Leroy C. Fouveaut C. Leclercq S. Jacquemont S. Boullay H. D. Lespinasse J. (2008). Biallelic mutations in the prokineticin-2 gene in two sporadic cases of Kallmann syndrome. Eur. J. Hum. Genet. 16, 865–868 10.1038/ejhg.2008.15 18285834

[B29] Li J. D. Hu W. P. Boehmer L. Cheng M. Y. Lee A. G. Siegel J. M. (2006). Attenuated circadian rhythms in mice lacking the prokineticin 2 gene. J. Neurosci. 26, 11615–11623 10.1523/JNEUROSCI.4727-05.2006 17093083 PMC2713041

[B30] Li J. D. Hu W. P. Zhou Q. Y. (2012). The circadian output signals from the suprachiasmatic nuclei. Prog. Brain Res. 199, 119–127 10.1016/B978-0-444-59427-3.00028-9 22877662

[B31] Li M. Bullock C. M. Knauer D. J. Ehlert F. J. Zhou Q. Y. (2001). Identification of two prokineticin cDNAs: recombinant proteins potently contract gastrointestinal smooth muscle. Mol. Pharmacol. 59, 692–698 11259612 10.1124/mol.59.4.692

[B32] Lin D. C. Bullock C. M. Ehlert F. J. Tian H. Zhou Q. Y. (2002). Identification and molecular characterization of two closely related G protein-coupled receptors activated by prokineticins/endocrine gland vascular endothelial growth factor. J. Biol. Chem. 277, 19276–19280 10.1074/jbc.M210611200 11886876

[B33] Martin C. Balasubramanian R. Dwyer A. A. Au M. G. Sidis Y. Kaiser U. B. (2010). The role of the prokineticin 2 pathway in human reproduction: evidence from the study of human and murine gene mutations. Endocr. Rev. 32, 225–246 10.1210/er.2010-0007 21037178 PMC3365793

[B34] Masuda Y. Takatsu Y. Terao Y. Kumano S. Ishibashi Y. Suenaga M. (2002). Isolation and identification of EG-VEGF/prokineticins as cognate ligands for two orphan G-protein-coupled-receptors. Biochem. Biophys. Res. Commun. 293, 396–402 10.1016/S0006-291X(02)00239-5 12054613

[B35] Matsumoto S.-I. Yamazaki C. Masumoto K.-H. Nagano M. Naito M. Soga T. (2006). Abnormal development of the olfactory bulb and reproductive system in mice lacking prokineticin receptor PKR2. Proc. Natl. Acad. Sci. U.S.A. 103, 4140–4145 10.1073/pnas.0508881103 16537498 PMC1449660

[B36] Mollay C. Wechselberger C. Mignogna G. Negri L. Melchiorri P. Barra D. (1999). Bv8, a small protein from frog skin and its homologue from snake venom induce hyperalgesia in rats. Eur. J. Pharmacol. 374, 189–196 10.1016/S0014-2999(99)00229-0 10422759

[B37] Monnier C. Dodé C. Fabre L. Teixeira L. Labesse G. Pin J.-P. (2009). PROKR2 missense mutations associated with Kallmann syndrome impair receptor signalling activity. Hum. Mol. Genet. 18, 75–81 10.1093/hmg/ddn318 18826963 PMC3298864

[B38] Naftolin F. Harris G. W. Bobrow M. (1971). Effect of purified luteinizing hormone releasing factor on normal and hypogonadotropic anosmic men. Nature 232, 496–497 10.1038/232496a0 4937217

[B39] Ng K. L. Li J.-D. Cheng M. Y. Leslie F. M. Lee A. G. Zhou Q.-Y. (2005). Dependence of olfactory bulb morphogenesis on prokineticin 2 signaling. Nature 308, 1923–1927 10.1126/science.1112103 15976302

[B40] Pinto G. Abadie V. Mesnage R. Blustajn J. Cabrol S. (2005). CHARGE syndrome includes hypogonadotropic hypogonadism and abnormal olfactory bulb development. J. Clin. Endocrinol. Metab. 90, 5621–5626 10.1210/jc.2004-0963 16030162

[B41] Pitteloud N. Zhang C. Pignatelli D. Li J.-D. Raivio T. Cole L. W. (2007). Loss-of-function mutation in the prokineticin 2 gene causes Kallmann syndrome and normosmic idiopathic hypogonadotropic hypogonadism. Proc. Natl. Acad. Sci. U.S.A. 104, 17447–17452 10.1073/pnas.0707173104 17959774 PMC2077276

[B42] Prosser H. M. Bradley A. Chesham J. E. Ebing F. J. P. Hastings M. H. Maywood E. S. (2007). Prokineticin receptor 2 (Prokr2) is essential for the regulation of circadian behaviour by suprachiasmatic nuclei. Proc. Natl. Acad. Sci. U.S.A. 104, 648–653 10.1073/pnas.0702093104 17202262 PMC1761911

[B43] Raivio T. Avbelj M. McCabe M. J. Romero C. J. Dwyer A. A. Tommiska J. (2012). Genetic overlap in Kallmann syndrome, combined pituitary hormone deficiency, and septo-optic dysplasia. J. Clin. Endocrinol. Metab. 97, E694–E699 10.1210/jc.2011-2938 22319038 PMC3319178

[B44] Raivio T. Sidis Y. Plummer L. Chen H. Ma J. Mukherjee A. (2009). Impaired fibroblast growth factor receptor 1 signaling as a cause of normosmic idiopathic hypogonadotropic hypogonadism. J. Clin. Endocrinol. Metab. 94, 4380–4390 10.1210/jc.2009-0179 19820032 PMC2775659

[B45] Reynaud R. Jayakody S. A. Monnier C. Saveanu A. Bouligand J. Guedj A. M. (2012). PROKR2 variants in multiple hypopituitarism with pituitary stalk interruption. J. Clin. Endocrinol. Metab. 97, 1068–1073 10.1210/jc.2011-3056 22466334

[B46] Sarfati J. Guiochon-Mantel A. Rondard P. Arnulf I. Garcia-Pineiro A. Wolczynski S. (2010). A comparative phenotypic study of Kallmann syndrome patients carrying monoallelic and biallelic mutations in the prokineticin 2 or prokineticin receptor 2 genes. J. Clin. Endocrinol. Metab. 95, 659–969 10.1210/jc.2009-0843 20022991

[B47] Schwanzel-Fukuda M. Pfaff D. W. (1989). Origin of luteinizing hormone-releasing hormone neurons. Nature 338, 161–164 10.1038/338161a0 2645530

[B48] Schweitz H. Pacaud P. Diochot S. Moinier D. Lazdunski M. (1999). MIT1, a black mamba toxin with a new and highly potent activity on intestinal contraction. FEBS Lett. 461, 183–188 10.1016/S0014-5793(99)01459-3 10567694

[B49] Sinisi A. A. Asci R. Bellastella G. Maione L. Esposito D. Elefante A. (2008). Homozygous mutation in the prokineticin-receptor 2 gene (Val274Asp) presenting as reversible Kallmann syndrome and persistent oligozoospermia: case report. Hum. Reprod. 23, 2380–2384 10.1093/humrep/den247 18596028

[B50] Soga T. Matsumoto S. Oda T. Saito T. Hiyama H. Takasaki J. (2002). Molecular cloning and characterization of prokineticin receptors. Biochim. Biophys. Acta 1579, 173–179 10.1016/S0167-4781(02)00546-8 12427552

[B51] Sykiotis G. P. Plummer L. Hughes V. A. Au M. Durrani S. Nayak-Young S. (2010). Oligogenic basis of isolated gonadotropin-releasing hormone deficiency. Proc. Natl. Acad. Sci. U.S.A. 107, 15140–15144 10.1073/pnas.1009622107 20696889 PMC2930591

[B52] Teixeira L. Guimiot F. Dodé C. Fallet-Bianco C. Millar R. P. Delezoide A.-L. (2010). Defective migration of neuroendocrine GnRH cells in human arrhinencephalic conditions. J. Clin. Invest. 120, 3668–3672 10.1172/JCI43699 20940512 PMC2947242

[B53] Tornberg J. Sykiotis G. P. Keefe K. Plummer L. Hoang X. Hall J. E. (2011). Heparan sulfate 6-O-sulfotransferase 1, a gene involved in extracellular sugar modifications, is mutated in patients with idiopathic hypogonadotropic hypogonadism. Proc. Natl. Acad. Sci. U.S.A. 108, 11524–11529 10.1073/pnas.1102284108 21700882 PMC3136273

[B54] Wechselberger C. Puglisi R. Engel E. Lepperdinger G. Boitani C. Kreil G. (1999). The mammalian homologues of frog Bv8 are mainly expressed in spermatocytes. FEBS Lett. 462, 177–181 10.1016/S0014-5793(99)01473-8 10580115

[B55] Young J. Metay C. Bouligand J. Tou B. Francou B. Maione L. (2012). SEMA3A deletion in a family with Kallmann syndrome validates the role of semaphorin 3A in human puberty and olfactory system development. Hum. Reprod. 27, 1460–1465 10.1093/humrep/des022 22416012

[B56] Zhang C. Ng K. L. Li J.-D. He F. Anderson D. J. Sun Y. E. (2007). Prokineticin 2 is a target gene of proneural basic helix loop-helix factors for olfactory bulb neurogenesis. J. Biol. Chem. 282, 6917–6921 10.1074/jbc.M703500200 17259180

